# Are we underestimating pathological fracture risk in malignant bone lesions of the proximal humerus?

**DOI:** 10.1007/s00256-025-04875-9

**Published:** 2025-01-18

**Authors:** Wolfram Weschenfelder, Friederike Weschenfelder, Christian Spiegel, Karin Gabriela Schrenk, Gunther Olaf Hofmann

**Affiliations:** 1 Department of Trauma, Hand and Reconstructive Surgery, University Hospital Jena, Am Klinikum 1, 07747 Jena, Germany; 2 Department of Obstetrics, University Hospital Jena, Jena, Germany; 3 Department of Hematology and Internal Oncology, Clinic of Internal Medicine II, University Hospital Jena, Jena, Germany

**Keywords:** Cancer, Metastases, Metastatic bone disease, Pathological fracture, Staging, Humerus

## Abstract

**Objective:**

This study is aimed at evaluating the distribution of metastatic bone disease (MBD), with a particular focus on the humerus, and its association with pathological fractures. Factors for contributing to the underestimation of fracture risk were assessed, including their impact on surgical management.

**Materials and methods:**

We retrospectively reviewed patient records of patients undergoing surgical treatment for MBD at our institution between 2005 and 2023. The analysis included factors such as medical history, tumour type, metastatic status, surgical method, lesion location, and imaging. The images of local and staging studies (CT chest/abdomen/pelvis, CT skeleton body, bone scan, PET/CT) were reviewed by two observers. Group comparisons were made based on lesion localisation.

**Results:**

The two most affected bone regions were the proximal femur (39.4%), followed by the proximal humerus (13.5%). Lesions of the proximal humerus were significantly more likely to be associated with pathological fractures compared to those of the proximal femur and other localisations (*p* < 0.01). Identified potential causes include less frequent depiction of the proximal humerus during staging (29% vs. 79% and 51%; *p* < 0.01) and overall lower Mirel’s scores despite the number of fractures (8 vs. 10 and 9; *p* < 0.01).

**Conclusion:**

Metastatic bone disease (MBD) in the proximal humerus is less frequently captured in current staging imaging, particularly CT chest/abdomen/pelvis. Additionally, fracture prediction using Mirel’s scoring often underestimates the actual risk. Staging investigations should include this region more comprehensively, and even when correctly imaged, better tools are needed to evaluate bone metastases effectively.

## Introduction

The number of patients living with metastatic bone disease (MBD) is rising, driving up healthcare costs and requiring greater multidisciplinary collaboration and specialised orthopaedic oncology knowledge [1–5]. With improved prognosis and earlier detection, MBD is increasingly treated curatively, often with wide resection, rather than with palliative approaches such as prophylactic stabilisation or fracture fixation [6]. Even in palliative situations, several studies indicate that pathological fractures increase complication rates and healthcare costs while also negatively impacting patient prognosis [7–9].

Improving MBD management faces challenges, especially in enhancing early and accurate detection. Current European Society for Medical Oncology (ESMO) guidelines recommend CT of chest/abdomen and bone scans for staging metastatic breast cancer [10] while staging for renal cell carcinoma includes CT of chest/abdomen/ pelvis but excludes bone scans [11].

**Fig. 1:**
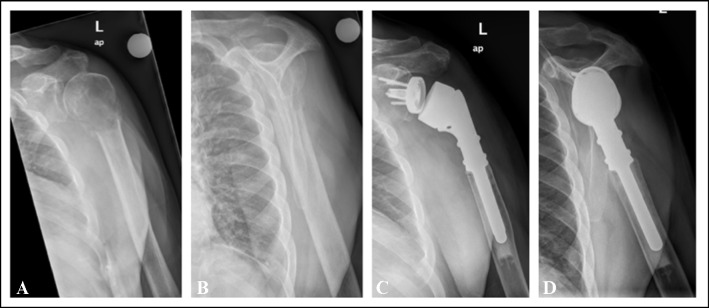
Singular bone metastasis of renal cell carcinoma in a 68-year-old patient, presenting as the primary clinical manifestation of the cancer initially treated as simple shoulder pain. **A**,** B** Radiograph images (anteroposterior and lateral views) of the shoulder and humerus demonstrate a pathological fracture through a subcapital osteolytic lesion. **C**,** D** Postoperative radiograph images (anteroposterior and lateral views) of the shoulder and humerus following reverse proximal humerus replacement after curative intraarticular resection

After the femur, the humerus is the second most common bone in the extremities to develop bone metastases [12]. However, it receives significantly fewer points on Mirel’s score regarding fracture risk (1 point versus 2 or 3 points, depending on the specific femoral location). Additionally, shoulder pain is common, making it challenging to distinguish between tumour-related pain—an additional parameter of Mirel’s score—and other types of pain [13] (see Fig. 1). Mirel’s score for upper limb lesions has been deemed valid and reproducible by Evans et al. despite a sensitivity of only 14.5% [14]. Recently, modifications to the evaluation criteria, including the addition of new variables and the use of AI tools, have been proposed [15–18].

The primary objective of this study was to assess the distribution of bone metastases in the body and evaluate how this correlates with pathological fractures, particularly concerning the humerus. Additional secondary outcomes aimed to identify potential reasons for the suspected neglect, such as poorer prognostic markers like metastatic status, staging diagnostic, or the type of surgery performed.

## Materials and methods

### Study population

**Fig. 2:**
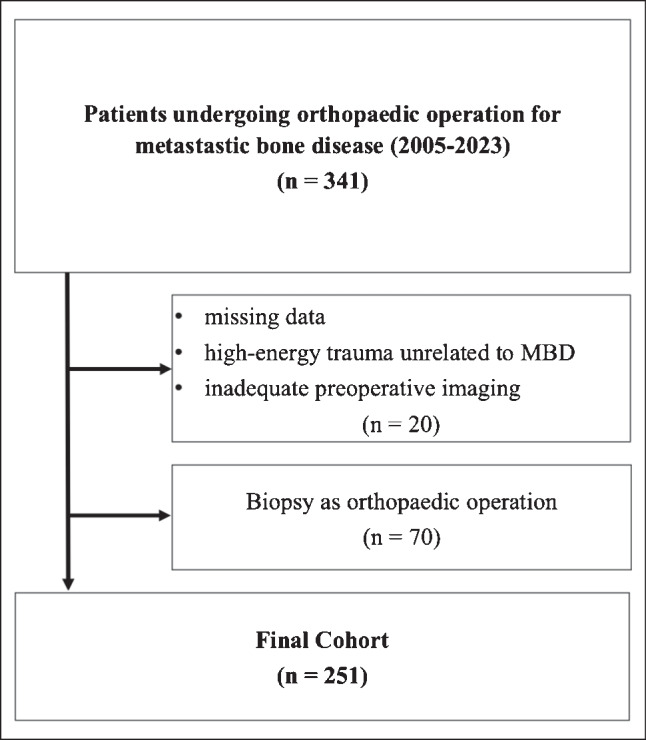
Flowchart of study population

We retrospectively reviewed the records of patients who underwent surgical treatment for metastatic bone disease, excluding cases involving spinal metastases, between January 2005 and December 2023 at our university hospital. The review was conducted in July 2024. Haematological disorders, such as lymphoma and multiple myeloma, were included in the analysis because their imaging, fracture risk assessment, and surgical treatment are comparable to those for MBD from visceral cancers. Of the 341 patients eligible for inclusion, 90 were excluded due to incomplete data, high-energy trauma unrelated to MBD, biopsy as the only procedure, or inadequate preoperative imaging. Consequently, 251 patients were included in the final analysis (see Fig. 2). This research was approved by the ethics committee of the authors’ affiliated institution (2023/3080-Daten).

### Data collection and statistical analysis

Patient characteristics, history, pathology, and imaging results were retrieved from patient records. Mirel’s scoring of x-ray imaging was independently performed by two experienced orthopaedic oncology consultants. The long bones were segmented according to the AO classification into proximal end segment, proximal diaphyseal segment, middle diaphyseal segment, distal diaphyseal segment, and distal end segment. Proximal end segment and proximal diaphyseal segment were labelled ‘proximal’ in the further text. Metastatic disease was defined as either single, oligometastatic (2–5 bone metastasis), or polymetastatic (> 5 bone metastasis) as per staging imaging reports. Metastatic status was not evaluated in patients with lymphoma or multiple myeloma. Previous staging imaging was reassessed by two observers in cases of known malignancy. Treatment was deemed suboptimal if the operated bone lesion was not identified in the report, if Mirels’ scores exceeded 7 without a recommendation for orthopaedic consultation in the multidisciplinary team meeting, or if surgery was recommended but not performed, with no medical justification documented in the patient records.

Statistical analysis was performed with SPSS 29.0 (IBM©, New York, NY, USA). We included all patients with inclusion and without exclusion criteria in our analysis. The interobserver reliability of Mirel’s scores between the two observers was evaluated using the kappa coefficient, which yielded a value of 0.49, indicating moderate reliability. Discrepancies were resolved through consensus with the inclusion of a third observer. Categorial data were compared with Chi^2^ test. Continuous data showed no normal distribution so non-parametric tests had to be used to determine differences; Mann–Whitney *U*-test in case of two; Kruskal–Wallis test in case of more than two categories. For significant differences, Cramer’s V was used to calculate the effect size for ordinal data and Cohen’s r for metric data. The respective median with interquartile range is shown in the text. A *p* < 0.05 was considered statistically significant.

## Results

### Baseline characteristics

The study population consisted of 124 females and 127 males: age ranging from 35 to 91 years with a median of 67 years (IQR 59–74 years). There was no significant difference regarding age among genders (*p* = 0.52). In 62 cases, the treated bone lesion represented the first clinical manifestation of the tumour disease (referred to as ‘primary manifestation’), while in 189 cases, the malignant disease was already known. Further baseline characteristics at the time of bone lesion treatment are given in Table 1.

**Table 1:** Baseline characteristics at time of treatment of bone lesion (median with interquartile ranges presented for metric data and number of cases presented for nominal data)

	Proximal femur	Proximal humerus	Other localisation	*p*-values
Gender, male Gender, female	46 53	22 12	59 59	0.18
Age, < 61 Age, 61–70 Age > 71	33 33 33	7 10 17	31 40 47	0.43
Primary manifestation Known malignancy	27 72	10 24	25 93	0.46
Most recent staging imaging within 24 months before treatment of bone lesion				0.79
- No staging - CT chest/abdomen/pelvis - CT skeleton body - Bone scan - PET/CT	26 28 5 3 7	10 8 3 1 2	26 38 8 8 13	
- Lesion on staging - Lesion not on staging	34 9	4 10	34 33	** < 0.01**
- Correct treatment - Suboptimal treatment	23 11	1 3	27 9	0.05
Time after primary diagnosis of cancer (months)	11 (0–64)	5 (0–34)	27 (1–81)	0.05
Renal cell carcinoma Multiple myeloma Breast cancer Lung cancer Other cancers	18 22 26 11 22	9 8 1 6 10	24 22 21 14 37	0.23
Clinical presentation				** < 0.01**
- Pain - Unstable fracture - Swelling - Other	41 53 1 4	5 28 1 0	32 70 6 10	
Pathological fracture No pathological fracture	57 42	34 0	75 43	** < 0.01**
Mirel’s Score	10 (9–10)	8 (8–9)	9 (8–10)	** < 0.01**
Bone quality, mixed Bone quality, lytic	25 74	4 30	27 91	0.26
Singular metastatic Oligometastatic Polymetastatic	7 12 56	4 3 17	21 12 58	0.23
Visceral metastases No visceral metastases	41 34	13 11	42 49	0.51
Wide resection Intralesional operation	17 82	7 27	29 89	0.41

**Fig. 3:**
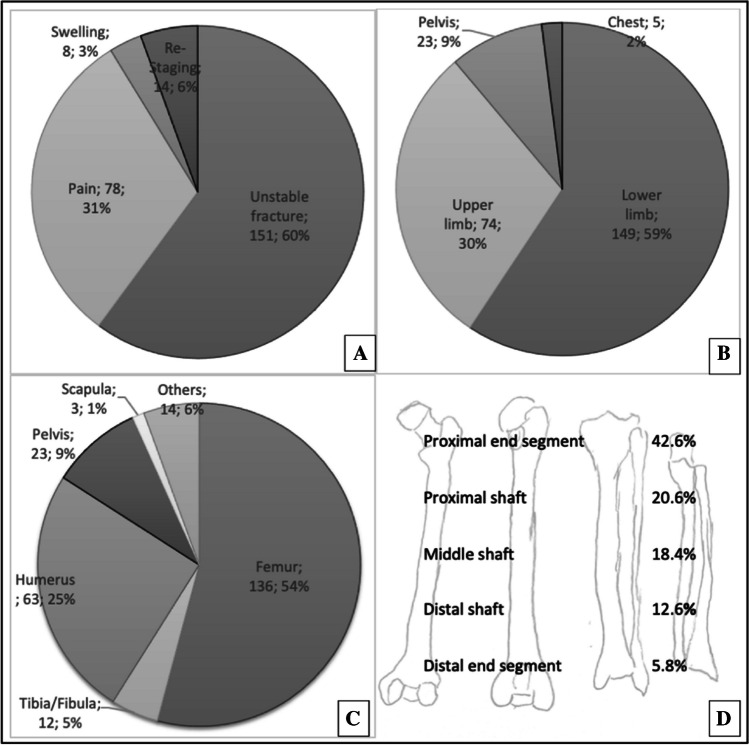
Clinical presentation (**A**), anatomic region (**B**), affected bone (**C**), and distribution of relevant bone lesion within the long tubular bones (**D**)

Figure 3 illustrates the reasons for imaging, the distribution of bone lesions within the body, and the most common bones and segments of long tubular bones affected. In 15 cases, the fracture presented clinically as pain but was not unstable, bringing the total number of pathological fractures to 166 (66.1%). The distribution of lesions across different bones and segments shows that 99 (39.4%) of all relevant bone metastases were located in the proximal third of the femur and 34 (13.5%) in the proximal humerus.

### Correlation of localisation of MBD and pathological fracture

**Fig. 4:**
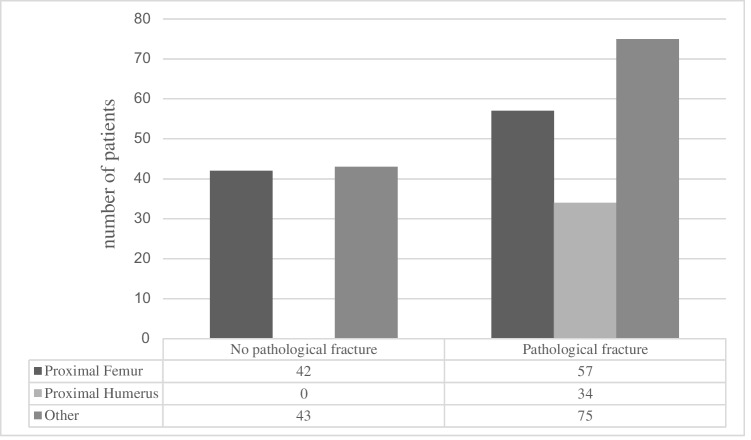
Distribution of patients presenting with pathological fractures among the most common sites of bone metastases

**Fig. 5:**
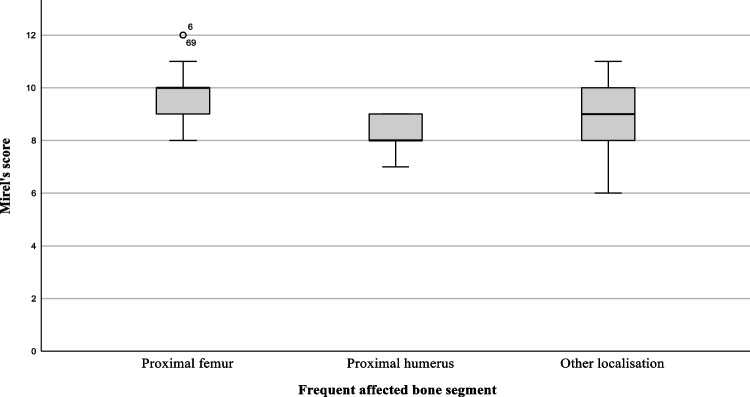
Boxplots of Mirels’ scores of affected bone regions

Figure 4 clearly shows that proximal humerus lesions are typically operated on for pathological fractures, whereas other locations tend to be treated earlier (*p* < 0.01; Cramer’s V = 0.29; see Fig. 4). The same applies to the reasons for orthopaedic consultation, as shown in Table 1, which illustrates a clear difference in the distribution of patients with pain and unstable fractures. In contrast, as expected, Mirels’ scores were significantly higher for lesions in the lower extremities (*p* < 0.01; see Fig. 5). The respective effect sizes were *r* = 0.57 (proximal femur – proximal humerus), *r* = 0.41 (proximal femur – other), and *r* = 0.18 (proximal humerus – other). There was no difference between the groups in terms of underlying tumour type, metastatic load, and the presence of visceral metastases as prognostic factors or the proportion of curative metastatic resections that were performed. The rate of new diagnoses and the time since the initial diagnosis of known tumour diseases also showed no significant differences between the groups (see Table 1).

### Potential influence of staging of MBD

Lesions of the proximal humerus were less likely to be detected using standard staging diagnostics (*p* < 0.01, Cramer’s V = 0.34). There was no difference in the diagnostic modality used across bone regions (*p* = 0.79). Additionally, there was no statistically significant difference in the quality of assessment according to existing scoring systems between the proximal femur, proximal humerus, and other locations (*p* < 0.05).

## Discussion

**Fig. 6:**
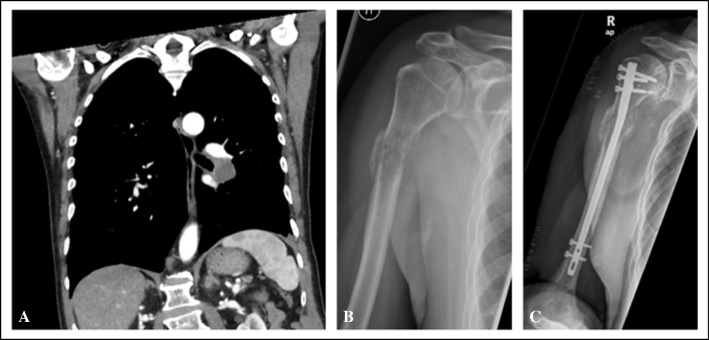
Bone lesion undetected during staging in a 71-year-old patient with lung cancer, presenting with a pathological proximal humerus fracture 5 months post-diagnosis. **A** Coronal CT of the chest, abdomen, and pelvis was performed 1 month prior for staging, showing the cranial extension of the scan without evidence of the lesion. **B** Anteroposterior radiograph image of the proximal humerus and shoulder demonstrating a pathological fracture caused by an osteolytic bone lesion. **C** Postoperative anteroposterior radiograph image of the humerus following closed reduction and fixation with a proximal humerus nail

In this retrospective study, we confirmed that proximal humerus lesions are typically treated orthopaedically when fractured, while lesions in other locations are often addressed earlier (*n* = 251). We found that this delay is not solely due to significantly lower Mirels’ scores for upper limb lesions; most proximal humerus lesions were not captured by staging imaging in patients with known cancers (see Fig. 6).

This is the first study to focus on the two most common sites of metastatic bone disease (MBD) in the extremities, specifically examining the reasons for the inadequate identification and stratification of MBD in the proximal humerus. In a multivariate analysis of 245 patients with MBD and 126 pathological fractures, Crenn et al. identified localisation in the upper extremity as a risk factor for pathological fractures, though they did not provide a cause [17]. This effect was also observed in our study group, where all lesions of the proximal humerus undergoing orthopaedic surgery were fractured (*n* = 34).

It was notable that there was a significant difference in the depiction of bone lesions among patients with known tumour disease across the staging examinations performed. Over 75% of treated MBDs at the proximal femur were detected, compared to approximately 50% of MBDs in other locations, and only 29% of lesions at the proximal humerus. The effect size was moderate, with a Cramer’s V of 0.34. This finding has not previously been described. The retrospective evaluation of treatment based on staging imaging showed no significant difference in quality regarding these locations; however, with only four proximal humeral lesions depicted, the statistical reliability of these figures is very limited.

No differences were observed between the groups in metastatic load, presence of visceral metastases, or surgical methods, suggesting that a substantial proportion of patients in the proximal humerus group also underwent metastasectomy with curative intent. Poorer imaging and the associated fracture risk at the proximal humerus pose significant challenges, as fractures complicate curative resections by opening compartments and potentially spreading tumour cells, often requiring more extensive surgery. This can adversely affect postoperative function and potentially prognosis, even with curative resection, as demonstrated by Saglam et al. and Salunke et al. for osteosarcomas [19, 20]. Given the similar surgical approach for bone metastases, these findings are relevant and, for example, suggest reconsidering the limitation of staging beyond chest CT in renal cell carcinoma patients only when clinically indicated [11, 21]. The number of undetected cases where lesions went unnoticed until a fracture occurred remains unclear. Even in palliative settings, prophylactic stabilisation before a fracture occurs is preferable, as it is more cost-effective and pathological fractures have limited healing potential, negatively impacting the patient’s quality of life [7, 22]. Therefore, early detection of MBD is valuable in these cases as well.

We were also able to confirm a known contributing factor: Mirels’ scores are significantly lower at the proximal humerus than at other sites, despite all patients in our cohort experiencing fractures there. Both Hoban et al. and Tat et al. have attempted to address this issue by adjusting Mirel’s score, either by lowering the cut-off or adding a cortical breach factor [15, 16]. The high effect size shown in our study, particularly in comparison to the proximal femur (*r* = 0.57), confirms the significantly different assessment of fracture risk for lesions in the proximal humerus. However, the assessment of fracture risk at the proximal femur itself has also recently been questioned, with a modified Mirel’s score proposed and validated using finite element models. This adjusted score identifies the subtrochanteric-diaphyseal region of the proximal femur as significantly more at risk of fracture [23, 24]. Additionally, the initial Mirel’s scoring was validated in a small population, and all bone metastases in this group were treated with radiation therapy. Overall, assessing fracture risk using only Mirel’s score now seems overly simplistic, and further efforts are needed to enhance predictive accuracy by integrating CT imaging and AI methods [18].

### Strength and limitations

A strength of this study is the relatively large patient cohort for a single-centre study, which ensures uniform treatment by the same team following consistent principles. However, the study is limited by the variability in tumour types and corresponding treatments, the extended observation period, and its retrospective design. Additionally, there is potential bias in Mirel’s scoring, as the analysis included only cases that underwent surgical intervention.

### Conclusion

The two most common regions for orthopaedically relevant bone metastases in the extremities are the proximal femur and the proximal humerus. Lesions of the proximal humerus are less frequently captured in standard staging diagnostics and receive lower scores in Mirel’s scoring. As a result, these lesions often go unnoticed until fractures occur. Therefore, staging exams should more effectively cover this region, and fracture risk assessment methods for bone metastases require revision.

## Data Availability

The data presented in this study are not publicly available but available on request from the corresponding author. The data are not publicly available due to privacy and ethical restrictions.

## Abbreviations

AI: Artificial intelligence
CT: Computed tomography
ESMO: European Society for Medical Oncology
IQR: Interquartile range
MBD: Metastatic bone disease
PET/CT: Positron emission tomography/computed tomography
